# Career intentions and perceptions of general practice on entry to medical school: baseline findings of a longitudinal survey at three UK universities

**DOI:** 10.3399/BJGPO.2021.0120

**Published:** 2021-11-10

**Authors:** Richard Darnton, Efthalia Massou, James Brimicombe, John Kinnear, Roger Tisi, Alys Burns, Diana F Wood, Paul O Wilkinson

**Affiliations:** 1 Department of Public Health and Primary Care, University of Cambridge, Cambridge, UK; 2 School of Medicine, Anglia Ruskin University, Cambridge, UK; 3 Norwich Medical School, University of East Anglia, Norwich, UK; 4 School of Clinical Medicine, University of Cambridge, Cambridge, UK

**Keywords:** career choice, intention, medical schools, medical students, perception, primary healthcare, prospective studies, general practice

## Abstract

**Background:**

Medical graduates from the universities of Oxford and Cambridge have a lower intention to become GPs compared with other UK medical graduates. It is not clear to what extent this difference is present on admission to medical school.

**Aim:**

To compare the career intention and influencing factors of students on admission to different UK medical schools.

**Design & setting:**

First year of a 6-year prospective cohort study of medical students admitted in autumn 2020 to the three East of England medical schools: University of East Anglia (UEA), University of Cambridge (UOC), and Anglia Ruskin University (ARU).

**Method:**

An online survey instrument was administered at the beginning of the first year. This measured self-reported career interests and various influencing factors, including perceptions of general practice.

**Results:**

UOC students declared a lower intention to become a doctor, a higher likelihood of choosing careers in pathology and public health, and a much lower likelihood of becoming a GP than students of UEA or ARU (all at *P*<0.001). In all three schools, the phrases least associated with general practice were 'opportunities for creativity/innovation' and 'research/academic opportunities', whereas the phrases most associated with general practice were 'favourable working hours' and 'flexibility'. However, research/academic opportunities were far more important, and favourable working hours far less important, to UOC students (*P*<0.001 for both) than to students of UEA or ARU.

**Conclusion:**

UOC students’ lower intention to become a GP appears to be present on entry to medical school. This may be explained in part by these students placing a higher importance on research/academic opportunities, combined with the widely held perception that GP careers lack these opportunities.

## How this fits in

Medical school curricula are thought to influence intention to choose GP careers. Graduates of Oxford and Cambridge are known to have a lower intention to become GPs compared with those of other UK medical schools, but whether this is a function of their curricula is not known. This study's findings in Cambridge students suggest this difference is present on entry to medical school, and could be explained in part by a much greater desire for research/academic careers than among students of other medical schools. Positive curricular influences on the acceptability of GP careers may, therefore, have little effect in certain subgroups of medical students if perceptions around lack of research/academic opportunities in GP careers are not addressed. These findings support earlier calls to raise the profile of academic general practice among certain groups of medical students.

## Introduction

There is an imperative on UK medical schools to produce future GPs^1,2^ and UK medical schools vary in the proportion of their graduates who enter GP training.^3,4^ Historically, the universities of Oxford and Cambridge (collectively termed ‘Oxbridge’) are the furthest outliers in this regard, with very low numbers of their graduates entering GP training.^5^ There is currently a lack of evidence regarding the reasons for this discrepancy, and its investigation may shed further light on factors influencing medical student career choice.

In 2004, Goldacre *et* *al* ^6^ demonstrated that medical students graduating from Oxbridge report significantly lower interest in following a career in general practice compared with those from other UK medical schools, but this study only contained minimal exploration of possible influencing factors. Since then, there have been a number of cross-sectional surveys investigating medical student career preferences and self-reported influencing factors,^7–11^ and a few of these^7,10^ have explored the degree to which medical students perceive GP careers as possessing these factors. However, none of these surveys have included measures of intolerance of uncertainty,^12^ which is significant, given that problem-based learning (PBL) is characterised by a response to uncertainty,^13^ and medical schools that employ a PBL approach have been shown to produce more future GPs.^4^

Moreover, none of the above studies have explicitly investigated the differences in acceptability of GP careers between Oxbridge students and students at other medical schools. The findings of a recent survey of Oxford medical students^7^ suggested that they may not perceive GP careers as offering sufficient potential for status, research opportunity, and financial reward. However, this study did not compare with other medical schools, did not investigate attitudes at admission, and did not include intellectual stimulation or measures of intolerance of uncertainty as possible influencing factors.

While there is some evidence to suggest that level of exposure to primary care in a medical school curriculum is associated with the degree to which it produces future GPs,^3,4,14^ Cambridge does not appear to fit this hypothesis, because the amount of time spent on GP placements puts it in the top 20% of medical schools in the country in this regard.^15^ The available literature does not fully explore the degree to which differences in career preferences and influencing factors are fixed on admission, nor the degree to which they change over time with exposure to the curriculum.

Therefore, a longitudinal survey was designed to compare over time the career drivers and perceptions of Cambridge medical students with those of two other medical schools that also provide high exposure to general practice. This article presents baseline results from the first year of that longitudinal study, principally looking at differences between cohorts shortly after admission to medical school.

## Method

### Study population

The cohort of medical students who commenced studies at three UK universities in autumn 2020 were included in the survey. These three medical schools are described below.

#### University of East Anglia

UEA has a 5-year PBL curriculum with vertical integration of clinical experience and high levels of exposure to GP-led teaching and primary care placements from year 1. Students spend at least 95 days of the course on GP placement.

#### Anglia Ruskin University

The ARU course has a 5-year horizontally integrated, systems-based curriculum with vertical integration of clinical experience and high levels of exposure to GP-led teaching and primary care placements from year 1. Students spend at least 60 days of the course on GP placement.

#### University of Cambridge

The first 3 years of the UOC curriculum comprise lecture and practical courses in core medical science disciplines with limited clinical exposure. The majority of clinical teaching and experience occurs in years 4–6 and includes high levels of exposure to primary care placements and GP-led seminars. Students spend at least 84 days of the course on GP placement.

### Sample and data

All first-year medical students at UOC (*n* = 327), ARU (*n* = 120), and UEA (*n* = 231) were asked to complete the questionnaire provided electronically via the Qualtrics survey platform. The survey took place between 5 October 2020 and 28 February 2021. The survey instrument is available to view in Supplementary Box 1. Data pre-processing and analysis were conducted via Stata (version 15.1).

### Measures

The survey was designed to assess students' expressed career intentions, drivers, perceptions, and potential influencing factors as they progress through their course. The items measured were based to a large part on the themes (although not the exact design) of the Royal College of General Practitioners’ *Destination GP* ^10^ questionnaire, as well as some additional themes.

The questionnaire asked students to rate the following:

- the importance they placed on a selection of career factors (using an electronic Likert scale 0–100)
- the degree to which they perceived general practice as possessing these factors (using an electronic Likert scale 0–100)
- their intention to be a doctor on completing their medical degree
- their likelihood of choosing a GP career
- their likelihood of choosing each of the following career options: anaesthetics, dermatology, emergency medicine, geriatrics, hospital medicine, obstetrics and gynaecology, paediatrics, pathology, psychiatry, public health, radiology, surgery, a career outside of medicine
- how GPs and general practice have been represented in the media in the last 6 months
- their experience of general practice as a patient or relative of a patient
- their experience on GP placement
- the extent to which they have encountered negativity towards general practice from
  - academics, clinicians, and/or educational trainers (using an electronic Likert scale 0–100)
  - other medical students (using an electronic Likert scale 0–100)
  - family and friends (using an electronic Likert scale 0–100)

Other than where stated, the scale for all the above was –50 to +50, where 0 = neutral.

Yes/No questions were used to determine whether or not:

- so far in the course, they have had a positive role model who happens to be a GP
- so far in the course, they have encountered an academic who is a GP
- they have attended a student GP society event
- they have a friend or family member who is a GP

An abbreviated version of the Intolerance of Uncertainty Scale (IUS)^12^ was used in the survey, and demographic data were also collected. As this was a longitudinal cohort study, some of the questions were not relevant to students at the beginning of their first year; the responses to those questions were not analysed.

### Data analysis

For ease of interpretation of the results, the –50 to +50 scales were recoded into 0–100 (50 becoming the neutral value). The minimum and maximum values as well as the mean, median, standard deviation (SD), and the 25^th^ and 75^th^ percentile were used for the continuous variables, and the percentages with the corresponding frequencies were used for the categorical variables. Measurement of intolerance of uncertainty involved calculating the sum score of more than one item. The reliability of these latent scores was evaluated using Cronbach’s alpha statistic.

For categorical variables, χ^2^ tests were used to investigate the differences between the three schools. For continuous variables, the univariate relationships were examined using one-way analysis of variance (ANOVA) in cases where its assumptions were met; otherwise, the non-parametric alternative of Kruskal–Wallis was applied. The statistic and the corresponding *P* value of the test were reported, with 0.001 being considered the level of statistical significance.

## Results

### Sample: size, response rates, and demographic characteristics of responders

The sample consisted of 483 students: 253 in UOC; 79 in ARU; and 151 in UEA. The response rates were 77.37% (*n* = 253/327) for UOC, 65.83% (*n* = 79/120) for ARU, and 65.37% (*n* = 151/231) for UEA. Cumulative response rates over time for the three groups are presented in Supplementary Figure S1. Descriptive statistics for the demographic characteristics of the sample are available in Supplementary Table S1.

### Career intention

UOC students declared a significantly lower intention to become a doctor (H(2)= 39.74, *P*<0.001), and a much lower likelihood of becoming a GP (H(2) = 47.16, *P*<0.001), than students at UEA and ARU. They also declared a significantly greater likelihood of choosing each of the 'non-patient facing' career options offered: pathology (H(2) = 59.56, *P*<0.001), public health (H(2) = 22.35, *P*<0.001), and careers outside medicine (H(2) = 20.98, *P*<0.001). Descriptive statistics for all questions about career intention are presented in Table 1a (comparing the three groups) and Table 1b (totals for the whole sample).

**Table 1a.** Intention to become a doctor and likelihood of choosing different careers, disaggregated by medical school (0 = definitely not/highly unlikely; 100 = definitely yes/highly likely)

	Career intention	ARU						UEA						UOC						Kruskal–Wallis (H),(*P* value)
Mean (SD)		Min	25^th^ perc.	Median	75^th^ perc.	Max	Mean(SD)	Min	25^th^ perc.	Median	75^th^ perc.	Max	Mean(SD)	Min	25^th^ perc.	Median	75^th^ perc.	Max		
As a doctor	97.44 (8.26)	40	100	100	100	100	95.16 (13.41)	0	97	100	100	100	89.69(16.35)	11	85	98	100	100	H(2) = 39.74, *P*<0.001
As a GP	51.91 (28.46)	0	34	53	71	100	54.10 (28.09)	0	30	60	75	100	35.08 (26.78)	0	13	28	53	100	H(2) = 47.16, *P*<0.001
Anaesthetics	43.08 (28.26)	0	20	50	66	94	42.63 (28.17)	0	19	45	67	100	51.84 (25.41)	0	37	59	70	100	H(2) = 12.78, *P* = 0.002
Dermatology	46.71 (30.17)	0	21	50	62	100	44.98 (29.64)	0	20	50	70	100	41.51 (26.48)	0	20	40	60	100	H(2) = 2.28, *P* = 0.320
Emergency Medicine	53.67 (29.37)	0	31	58	78	100	60.01 (29.73)	0	41	65	82	100	55.93 (26.38)	0	38	62	75	100	H(2) = 4.13, *P* = 0.127
Geriatrics	27.47 (25.07)	0	0	20	50	86	30.92 (26.45)	0	7	28	50	100	33.16 (22.55)	0	17	34	50	92	H(2) = 5.09, *P* = 0.079
Hospital Medicine (the physicianly specialties)	66.01 (23.20)	0	55	70	80	100	65.22 (23.01)	0	50	70	80	100	69.64 (20.22)	0	58	72	84	100	H(2) = 3.04, *P* = 0.219
Obstetrics & Gynaecology	48.87 (29.17)	0	20	53	70	100	48.34 (30.34)	0	22	50	70	100	50.36 (28.45)	0	30	54	71	100	H(2) = 0.34, *P* = 0.843
Paediatrics	66.68 (29.91)	0	56	75	90	100	60.51 (33.08)	0	30	67	90	100	56.11 (28.17)	0	35	59	80	100	H(2) = 11.54, *P* = 0.003
Pathology	31.58 (26.94)	0	2	30	50	100	23.68 (24.66)	0	0	19	40	94	44.43 (26.13)	0	25	50	65	100	H(2) = 59.56, *P*<0.001
Psychiatry	43.20 (33.47)	0	4	41	69	100	48.45 (30.42)	0	20	55	70	100	41.43 (26.87)	0	19	42	62	98	H(2) = 5.99, *P* = 0.050
Public Health	29.06 (25.11)	0	0	29	50	100	30.44 (26.73)	0	4	25	50	95	41.92 (28.16)	0	17	41	62	100	H(2) = 22.35, *P*<0.001
Radiology	38.48 (29.96)	0	4	43	63	100	39.93 (28.57)	0	17	41	61	100	41.95 (26.15)	0	25	43	62	100	H(2) = 0.95, *P* = 0.619
Surgery	58.99 (33.48)	0	30	66	86	100	59.97 (32.11)	0	40	66	85	100	67.48 (28.19)	0	50	75	90	100	H(2) = 5.98, *P* = 0.05
Outside of medicine	15.16 (21.79)	0	0	1	28	75	21.91 (29.14)	0	0	5	45	100	28.75 (28.60)	0	1	20	51	100	H(2) = 20.98, *P*<0.001

SD standard deviation ARU Anglia Ruskin University UOC University of Cambridge UEA University of East Anglia perc. percentile

**Table 1b.** Intention to become a doctor and likelihood of choosing different careers, aggregated data (0 = definitely not/highly unlikely; 100 = definitely yes/highly likely).

	Total					
	Mean (SD)	Min	25^th^ perc.	Median	75^th^ perc.	Max
Career as a doctor	92.69 (14.73)	0	90	100	100	90
Career as a GP	43.78 (28.91)	0	20	41	75	20
Anaesthetics	47.53 (27.10)	0	24	50	70	100
Dermatology	43.45 (28.14)	0	20	46	63	100
Emergency Medicine	56.84 (28.00)	0	38	61	79	100
Geriatrics	31.53 (24.28)	0	10	30	50	100
Hospital Medicine (the physicianly specialties)	67.67 (21.68)	0	55	71	82	100
Obstetrics & Gynaecology	49.48 (29.12)	0	25	53	71	100
Paediatrics	59.22 (30.25)	0	36	65	85	100
Pathology	35.84 (27.42)	0	10	35	57	100
Psychiatry	43.91 (29.56)	0	18	48	67	100
Public Health	36.23 (27.83)	0	10	36	59	100
Radiology	40.75 (27.54)	0	19	42	62	100
Surgery	63.74 (30.55)	0	47	70	90	100
A career outside of medicine	24.39 (28.19)	0	0	10	50	100

SD standard deviation perc. percentile

### Importance of career factors and the degree to which they are associated with general practice

Table 2 presents descriptive statistics regarding the degree to which certain phrases were associated with general practice. For each of the three schools, the phrases least associated with general practice were 'opportunities for creativity/innovation' and 'research/academic opportunities' while the phrases most associated with general practice were 'favourable working hours' and 'flexibility'. In general, there were not statistically significant differences between the three schools in the extent to which they associated the different phrases with general practice. The one exception was research/academic opportunities, with students in UEA scoring this lower than the other two schools (*F* = 6.91, *P* = 0.001).

**Table 2.** Extent of associating certain words with general practice, in each school (0 = not at all; 100 = completely)

Variable	ARU					UEA					UOC					Total					Kruskal–Wallis (H),(*P* value)
	Mean (SD)	Min	25^th^ perc.	Median	75^th^ perc.	Mean (SD)	Min	25^th^ perc.	Median	75^th^ perc.	Mean (SD)	Min	25^th^ perc.	Median	75^th^ perc.	Mean (SD)	Min	25^th^ perc.	Median	75^th^ perc.	
Intellectual stimulation	58.28(25.57)	1	37	62	76	55.95 (24.81)	2	37	55	75	54.83(26.74)	0	35	55	75	55.74 (25.94)	0	36	57	75	H(2) = 1.35, *P* = 0.510
Favourable working hours	83.57(17.91)	9	75	89	100	79.99 (22.10)	3	70	85	100	77.68(23.89)	0	70	85	99	79.36(22.52)	0	70	86	100	H(2) = 2.61, *P* = 0.271
Flexibility	76.39(23.55)	10	65	85	97	72.28 (24.71)	2	60	78	92	69.07(25.40)	0	50	75	90	71.27 (24.99)	0	54	77	90	H(2) = 6.623, *P* = 0.037
Research/ academic	42.24(27.70)	0	17	46	62	29.92(24.54)	0	10	24	46	38.08(27.32)	0	16	35	58	36.21(26.87)	0	14	32	56	H(2) = 13.126, *P* = 0.001
Prestige/ status	49.53(27.66)	1	25	50	72	48.44(24.91)	0	30	50	66	48.11(25.21)	0	30	50	67	48.45(25.48)	0	30	50	68	H(2) = 0.144, *P* = 0.930
Team working	57.63(30.82)	0	30	60	87	51.05(26.88)	0	30	50	71	57.70(26.69)	0	36	58	79	55.61(27.58)	0	34	54	77	H(2) = 6.012, *P* = 0.049
Scientifically based	59.94(26.05)	1	42	60	80	58.11(26.89)	2	37	60	79	60.37(26.59)	0	41	62	81	59.59(26.56)	0	40	60	80	H(2) = 0.782, *P* = 0.676
Leadership	60.19(28.66)	0	35	62	85	55.25(26.63)	0	36	53	76	58.45(25.42)	0	40	60	76	57.73(26.36)	0	38	60	80	H(2) = 2.285, *P* = 0.304
Opportunities for creativity	37.75(26.12)	0	15	30	59	33.56(24.24)	0	14	30	50	35.63(25.02)	0	15	31	50	35.33(24.95)	0	15	30	50	H(2) = 1.241, *P* = 0.537

The maximum for all variables and schools was 100 and it is omitted from the table for space economy.

ARU Anglia Ruskin University SD standard deviation perc. percentile UOC University of Cambridge UEA University of East Anglia

Table 3 presents the scores for importance of certain career factors. For all three medical schools, the top two career factors rated as the most important were intellectual stimulation and team working. UOC students appeared to rate the importance of some factors quite differently to the UEA and ARU students, whose scores were more closely aligned. UOC students rated intellectual stimulation and research/academic opportunities significantly higher (H(2) = 25.4, *P*<0.001, and H(2) = 64.07, *P*<0.001 correspondingly), than those from the other two universities. They also rated flexibility significantly less important (H(2) = 15.13, *P*<0.001), and favourable working hours much less important (H(2) = 22.50, *P*<0.001) than those from the other two universities.

**Table 3.** Responses for 'Rate how important the following factors are to you when choosing your future career?' (0 = not at all; 100 = completely)

Variable	ARU					UEA					UOC					Total					Kruskal–Wallis (H),(*P* value)
	Mean (SD)	Min	25^th^ perc.	Median	75^th^ perc.	Mean (SD)	Min	25^th^ perc.	Median	75^th^ perc.	Mean (SD)	Min	25^th^ perc.	Median	75^th^ perc.	Mean (SD)	Min	25^th^ perc.	Median	75^th^ perc.	
Intellectual stimulation	79.11 (20.60)	0	70	81	96	77.91(17.8)	19	70	80	91	86(16.35)	0	79	90	100	82.34(17.94)	0	73	85	100	H(2)=25.4, *P*<0.001
Flexibility	68.20 (24.97)	15	50	73	90	67.26(24.24)	0	50	70	85	58.68(25.98)	0	40	60	77	62.92(25.62)	0	49	65	81	H(2) = 15.13, *P*<0.001
Opportunities for creativity	60.81 (25.91)	0	44	63	79	57.67(25.81)	0	39	57	75	65.2(25.95)	1	50	70	85	62.13(26.07)	0	47	65	82	H(2) = 10.03*P* = 0.007
Working hours	70.70 (24.05)	1	57	75	90	68.13(25.75)	0	50	71	88	57.49(27.72)	0	40	60	80	62.98(27.12)	0	46	68	82	H(2) = 22.50, *P*<0.001
Team working	77.75 (22.91)	0	66	81	100	74.66(22.58)	0	62	80	90	72.61(23.38)	0	60	76	90	74.09(23.08)	0	61	79	91	H(2) = 4.06,*P* = 0.131
Research/ academic opportunities	50.14 (26.72)	0	30	51	68	43.28(27.26)	0	20	45	62	65.3(26.19)	0	50	70	83	55.94(28.41)	0	35	60	79	H(2) = 64.07, *P*<0.001
Prestige/status	34.76 (27.53)	0	10	30	52	38.41(28.14)	0	15	31	59	41.76(28.08)	0	16	45	62	39.57(28.07)	0	15	40	60	H(2) = 4.51, *P* = 0.11
Opportunities for leadership	61.78 (27.17)	0	50	65	80	59.75(27)	0	40	64	80	61.02(25.72)	0	48	65	79	60.75(26.32)	0	43	65	80	H(2) = 0.57, *P* = 0.75

The maximum for all variables and schools was 100 and it is omitted from the table for space economy.

ARU Anglia Ruskin University UEA University of East Anglia UOC University of Cambridge perc. percentile SD standard deviation

**Figure 1.**
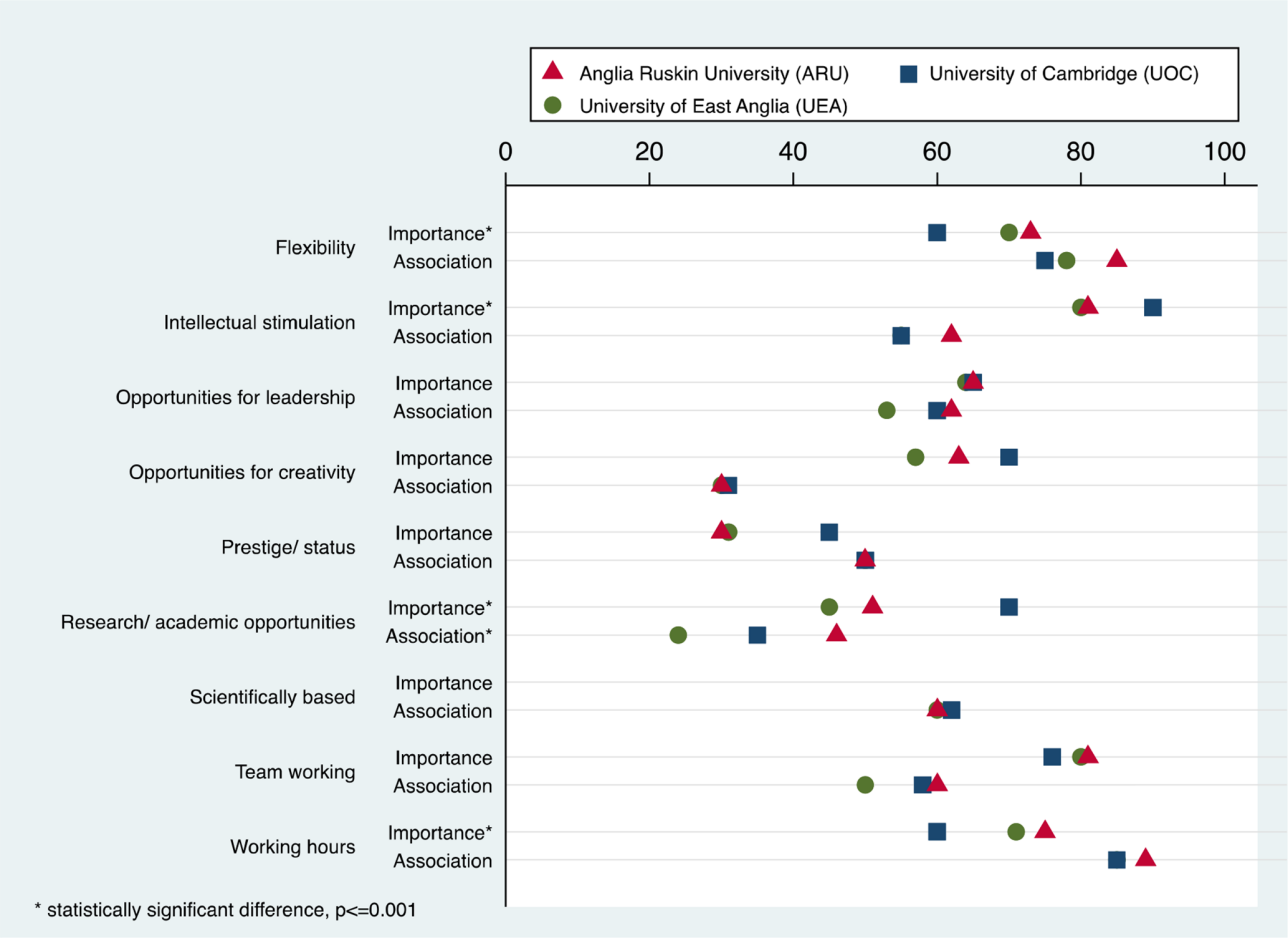
Importance of career factors and degree to which they were associated with general practice. Where only two shapes are evident this is because the third is obscured by another which has the same value.

Figure 1 illustrates differences between the three medical schools in terms of the importance students placed on certain career factors and the degree to which they associated these factors with general practice. It also demonstrates for each school where there is a mismatch between the perceived importance of a factor and its perceived association with general practice. Such mismatches appear to be most evident in the UOC group, especially for research/academic opportunities and favourable working hours.

### Intolerance of Uncertainty

A score was constructed as a sum of the eight items. The internal consistency of this measure was good in all schools (in UOC the Cronbach’s alpha was 0.76, while in ARU and UEA it was 0.79). It was found that intolerance of uncertainty was not significantly different between the three schools (F-test = 2.07, *P* = 0.13). A summary of the scores for the three medical schools is available in Supplementary Table S2.

### Other potential influencing factors (role models, encountering a GP academic, a GP friend/family member, quality of experience as a patient or relative, experiencing negativity from staff/students/family/media)

There was no statistically significant difference between the three groups in terms of:

- having friends or family members who were GPs (*n* = 70 [27.67%] in UOC; *n* = 28 [35.44%] in ARU; *n* = 51 [33.77%] in UEA; χ^2^ = 2.58; *P* = 0.27);
- perceived exposure to negativity about general practice from media (*F* = 1.44, *P* = 0.238), staff (H(2) = 5.66, *P* = 0.06), students (H(2) = 4.54, *P* = 0.10), family and friends (H(2) = 2.73, *P* = 0.25) (see Table 4); or
- the perceived quality of their experience of general practice as a patient or relative (H(2) = 3.37, *P* = 0.186) (see Table 4).

**Table 4.** Media representation of GPs/negativity from staff, students, family and friends/quality of experience of GP as a patient or relative (0 = extremely negatively/not at all/very negative; 100 = extremely positively/a great deal/very positive)

Variable	Anglia Ruskin University (ARU)				University of East Anglia (UEA)				University of Cambridge (UOC)				Total				Kruskal Wallis (H), (p-value)
	Mean (sd)	25^th^ perc.	Median	75^th^ perc.	Mean (sd)	25^th^ perc.	Median	75^th^ perc.	Mean (sd)	25^th^ perc.	Median	75^th^ perc.	Mean (sd)	25^th^ perc.	Median	75^th^ perc.	
Media *	54.81 (21.36)	40	50	71	58.64 (20.27)	41	61	73	55.51 (19.44)	40	58	70	56.37 (20.04)	40	60	71	F(2)=1.44, *P*=0.238
Negativity from academics, clinicians and/ or educational trainers	14.51 (23.19)	0	5	15	21.56 (24.94)	0	10	32	18.75 (24.31)	0	7	29	18.94 (24.39)	0	8	30	H(2)=5.66, *P*=0.06
Negativity from medical students	29.90 (29.95)	1	22	53	33.37 (28.30)	7	30	52	27.81 (27.33)	1	21	50	29.89 (28.13)	2	23	50	H(2)=4.54, *P*=0.10
Negativity from family and friends	21.68 (27.64)	0	8	30	27.77 (31.05)	1	15	57	27.34 (29.67)	1	13	50	26.55 (29.81)	1	11	50	H(2)=2.73, *P*=0.25
Experience of general practice as a patient or relative	65.66 (26.04)	50	70	83	70.64 (26.09)	58	80	75	68.32 (25.51)	51	75	89	68.61 (25.51)	50	77	89	H(2)=3.37, *P*=0.186

The minimum and the maximum for all variables and schools was 0 and 100 correspondingly, and it is omitted from the table for space economy.

* Denotes that ANOVA F test was done for this variable rather than Kruskal–Wallis test as data met its assumptions

ARU Anglia Ruskin University UEA University of East Anglia UOC University of Cambridge perc. percentile SD standard deviation

A much higher proportion of UEA and ARU students reported exposure to GP academics (*n* = 71 [89.87%] in ARU, *n* = 106 [70.20%] in UEA, *n* = 88 [34.78%] in UOC) and positive GP role models (*n* = 65 [82.87%] in ARU, *n* = 131 [86.75%] in UEA, *n* = 125 [40.41%] in UOC). Although statistical testing met the threshold for statistical significance (role model χ^2^ = 69.78, *P* = 0.000; GP Academic χ^2^ = 94.64, *P* = 0.000), whether this difference is of true significance is doubtful. This is because, as elaborated on in the discussion, the UOC survey reached a suitable response rate much earlier in the first year than the surveys in the other two schools. This will have resulted in a significantly longer exposure to the curriculum (and thus educators) in the ARU and UEA groups.

## Discussion

### Summary

On entry to medical school, UOC students declare a lower likelihood of choosing a GP career, or indeed any career in medicine, compared with UEA and ARU students. They also declare a higher interest in the following careers: pathology, public health, and careers outside medicine.

Perceptions of general practice were broadly similar across the three groups. The phrases most associated with general practice were 'favourable working hours' and 'flexibility', while the phrases least associated with general practice were 'opportunities for creativity/innovation' and 'research/academic opportunities'. However, UOC students differed significantly from ARU and UEA students in how important these factors were to them. In particular, UOC students rated research/academic opportunities as much more important to them, and favourable working hours as much less important to them, compared with ARU and UEA students. The most obvious mismatches between the perceived importance of a factor and its perceived association with general practice, therefore, appeared to occur in the UOC group, and were related to these two factors (Figure 1).

In terms of intolerance of uncertainty and the other potential influencing factors that were surveyed, differences between the groups were non-significant.

### Strengths and limitations

A limitation of the survey instrument was that only eight out of the 27 questions in the IUS^12^ were included. This decision was taken in light of the unfeasible length that the full tool would have added to the questionnaire. While there is a validated 12-item intolerance of uncertainty questionnaire available (IUS-12),^16^ many of the questions with most face validity for learners are not contained in the 12-item tool. It was, however, ensured that the eight questions chosen were equally balanced between the two subscales of the 27-point IUS tool ('uncertainty has negative behavioural and self-referent implications' and 'uncertainty is unfair and spoils everything').^17^ It was also demonstrated statistically that the shortened eight-item tool has good internal consistency. Nevertheless, further work is required to confirm its construct validity with regards to uncertainty in learning and in clinical decision making.

This first survey of the cohort study was purposed to measure student responses on entry to medical school to provide a baseline for future years, before exposure to the curriculum had a chance to take effect. However, after the survey opened on 5 October 2020, UOC, ARU, and UEA took different lengths of time to reach their respective final response rates of 77%, 66%, and 65% (see Supplementary Figure S1 for a graph of cumulative response rates by school). For example, UOC reached 60% by week 1, and ARU reached that level at week 9. UEA reached 40% by week 11 and 60% by week 21. This means that, for example, one-third of UEA responders will have had up to 3 months more exposure to the first-year curriculum before completing the survey. Finally, another limitation of this study may lie in the selection of 0.001 as significance level. Chosen to reduce type I errors resulting from multiple testing, this level could increase the chance of type II errors.

### Comparison with existing literature

It is already well documented that graduates of Oxford and Cambridge medical schools have a lower interest in GP careers compared with other UK medical schools.^6^ However, this study's results add to the literature by demonstrating that in Cambridge students, this difference appears to be present around the time of admission to medical school, rather than being solely attributable to subsequent influences.

Students’ perceptions of the nature of general practice were consistent with the results of previously published surveys.^7–10^ However, this study adds to the literature by comparing perceptions between the three groups. It is revealing to see that all three groups of students viewed general practice similarly for all but one factor (and even with that factor included, UOC was not the outlier). This suggests that Cambridge students’ lower interest in GP careers is not due to a different perception of the nature of general practice. Similarly, the data also suggest that this lower interest at admission does not appear to be related to any difference in those influencing factors that could act before medical school such as intolerance of uncertainty, exposure to negativity about general practice, quality of experience as a patient, or having friends or family members who are GPs.

A desire for intellectual stimulation is thought to be a very important factor influencing medical student subspecialty choice.^10,18^ The present study's results confirm this by demonstrating that it was the factor that all three groups of students rated most important to them. However, the data suggest that differing importance placed on certain career factors, when combined with commonly held perceptions of general practice, may provide a clue to Cambridge students’ lower interest in GP careers (see Figure 1). For example, while all three groups of students perceived general practice as possessing very low research/academic opportunities and highly favourable working hours, UOC students placed much greater importance on the former and much less importance on the latter, compared with students from the other medical schools.

### Implications for research and practice

A lower interest in primary care careers of Oxbridge medical students compared with those from other UK medical schools is well documented.^6^ However, this study is the first to explore the underlying reasons for this difference. Its findings show that for Cambridge students this difference exists at admission to medical school. The results suggest that this may be explained in part by the greater importance Cambridge students place on having a career with research/academic opportunities, combined with the widely held perception among medical students that general practice offers little in this regard. This adds weight to current calls to raise the profile of academic general practice among certain groups of medical students.^19,20^

Current thinking is that increasing the amount of exposure to general practice at medical school and reducing negativity from students, staff, and media is key to increasing medical student interest in GP careers.^3,10,14^ However, these findings suggest that further research is required to determine whether such interventions have the desired effect in subgroups of students, such as those at Cambridge who desire a career with research/academic opportunities. Given that the Cambridge curriculum contains a relatively high GP placement content, continuing this longitudinal study should help to answer this question. If the data on exit remain similar to that on entry, it will suggest that Cambridge students, and other similarly profiled students, may be impervious to such interventions without first addressing a broad perception of a lack of research/academic opportunities in primary care.
